# Technical Description of eHealth Tools in an Environmental Exposure Chamber: Implementation Study

**DOI:** 10.2196/71276

**Published:** 2025-08-26

**Authors:** Irene Garcia-Gutierrez, Irene Dueñas-Requena, Violeta Sanchez-Garcia, Cristiana Solorzano-Zepeda, Samuel Santos-Benito, Belen de la Hoz-Caballer, Dario Antolin-Amerigo

**Affiliations:** 1 Allergy Department, Marqués de Valdecilla University Hospital, Santander, Spain; 2 Instituto de Investigación Marqués de Valdecilla, Santander, Spain; 3 Faculty of Medicine, Universidad de Alcalá, Alcalá de Henares, Spain; 4 Allergy Department, Hospital Universitario Ramón y Cajal, M-607, km. 9, 100, Madrid, 28034, Spain, 34 913369072; 5 Technical School of Telecommunication Engineering, Universidad Politécnica de Madrid, Madrid, Spain; 6 Instituto Ramón y Cajal de Investigación Sanitaria, Madrid, Spain

**Keywords:** telemedicine, allergen challenge, respiratory allergy, remote symptom monitoring, REDCap, Power BI, allergic rhinitis, asthma, digital health, clinical data integration

## Abstract

**Background:**

Environmental exposure chambers (EECs) provide a controlled and reproducible setting for studying allergic rhinitis, allowing standardized exposure to aeroallergens. However, real-time symptom monitoring remains challenging, particularly as patients must be observed remotely while inside the chamber.

**Objective:**

This study aimed to design, implement, and evaluate an integrated eHealth system that leverages digital tools for data collection and real-time clinical monitoring during EEC exposures. The goal was to streamline workflow, enhance data reliability, and improve patient safety during allergen challenges.

**Methods:**

We conducted a prospective pilot study in a validated EEC at Ramón y Cajal University Hospital. A total of 34 provocation tests were conducted: 27 in patients with confirmed grass pollen allergy and 7 in nonallergic controls. All exposures took place outside the pollen season using standardized *Phleum pratense* (grass pollen) at concentrations of 940±100 grains/m³. Clinical data were collected using REDCap (Research Electronic Data Capture; Vanderbilt University), a secure electronic data capture platform. Surveys were automatically scheduled and included standardized instruments: Visual Analog Scale (VAS), Total Nasal Symptom Score (TNSS), Total Ocular Symptom Score (TOSS), asthma symptom questionnaires, peak expiratory flow (PEF), and peak nasal inspiratory flow (PNIF). Data were visualized in real time using Power BI dashboards (Microsoft Corp).

**Results:**

Among allergic patients, 85% had a positive response to the allergen exposure, with a median TNSS of 6 (IQR 6‐7), while controls showed no positive reactions. Mean exposure time for positive cases was 48.9 minutes (SD 28.3), as patients were withdrawn early upon reaching symptom thresholds. The REDCap system was configured to collect symptom surveys at 15-minute intervals, allowing up to 6 entries per patient during exposure, depending on clinical tolerance. This setup enabled consistent symptom monitoring and integration with Power BI for real-time visualization. All records were successfully synchronized between REDCap and Power BI. Informal feedback from medical staff highlighted improved workflow and usability.

**Conclusions:**

The integration of REDCap with Power BI enabled automated, real-time tracking of symptoms during EEC exposures. This eHealth solution enhanced clinical oversight, supported timely decision-making, and ensured patient safety. The platform provides a flexible and reproducible model for use in allergen exposure studies and other clinical settings requiring structured remote monitoring. Clinical response data are reported in a separate validation study.

## Introduction

Allergic rhinitis affects 1 in 5 people worldwide. Among other things, allergic rhinitis is associated with a decrease in overall quality of life and productivity loss. Although there are various treatment options, some patients remain undertreated or affected by drug side effects. Rhinoconjunctivitis and asthma in patients who are polysensitized to different aeroallergens constitute a challenge for their diagnosis and treatment, since there is a need to identify the relationship between the load of a specific allergen and patients’ symptoms, considering that, in some cases, pollination takes place at the same time of the year for different species. For this reason, a greater understanding of etiological agents and their pathological mechanisms may be effective in guiding a more precise treatment [1].

Environmental exposure chambers (EECs) are generally recognized as safe, effective, and suitable for conducting randomized clinical trials of new allergy treatments [2]. Since their introduction, EECs have provided a controlled setting for allergen exposure, ensuring reproducibility and safety. They have become increasingly important in clinical research, particularly in studies of allergic rhinitis, as they provide a fixed allergen concentration in a tightly controlled environment [3], and outcomes observed within EECs are comparable to those obtained in natural exposure settings, suggesting the usefulness of EECs in allergic rhinitis studies [4].

Mobile health (mHealth) has emerged as a transformative force in the health care industry, leveraging the power of mobile technology to enhance medical services and improve overall health outcomes. These devices have become an indispensable part of modern life, offering a variety of health care–related apps and services [5]. With the widespread adoption of smartphones and other portable devices, mHealth has enabled individuals to access health care resources, monitor their health, and receive medical guidance. Digital solutions are the new challenge of today’s fast-paced, changing, and volatile society.

Due to the characteristics of EECs, all monitoring of patient symptoms must be done remotely, as the patient remains inside the sealed environment during exposure. Real-time access to clinical data—such as symptom scores, positivity thresholds, and environmental variables—is essential to ensure both patient safety and the validity of the provocation. Without continuous monitoring, symptom escalation could go undetected, potentially compromising both participant well-being and study reliability.

## Methods

### Study Design

The exposures within the EEC were conducted for periods up to 90 minutes, scheduled outside the grass pollen season, to minimize the risk of priming phenomena. The design ensured that the responses observed during chamber exposures were attributable solely to the controlled allergen administration.

### Environmental Exposure Chamber

The EEC used in this study is a fully controlled, state-of-the-art facility located at the Ramón y Cajal University Hospital (RyCUH). The EEC has a total area of 15.60 m^2^ and is equipped to maintain stable temperature, relative humidity, and air pressure, ensuring homogeneous distribution of allergen particles through a calibrated air circulation system. The allergen administered was standardized grass pollen (*Phleum pratense*), aerosolized using a precision nebulizer at concentrations ranging between 940±100 grains/m^3^.

The EEC has undergone formal validation [6] for use in allergic respiratory studies, including verification of allergen distribution uniformity, reproducibility of symptom induction, and environmental stability, as described in García-Gutiérrez et al [6]. This validation process ensures that the chamber provides consistent and controlled allergen exposure conditions across sessions. Such standardization is essential for both patient safety and scientific reproducibility in allergen challenge studies.

### Study Population

The study population comprised 31 volunteers, including 25 patients with confirmed grass pollen allergy (cases) and 6 non–grass-allergic controls. Participants had a mean age of 31 (SD 10.6) years, and the sex distribution was 22 women and 9 men. Among the allergic individuals, 8 (32%) had mild asthma as a comorbidity. All cases were sensitized to *Phleum pratense*, confirmed by skin prick tests (mean wheal diameter 8.1 mm, SD 3.4 mm) or specific immunoglobulin E levels (median 20.3 kU/L, IQR 4.9‐42.4 kU/L). Controls included 3 individuals without any sensitization and 3 with sensitization to other aeroallergens (*Olea europaea* and *Cupressaceae* or dust mites). None of the control participants developed symptoms during pollen exposure. All participants were free of allergy-specific treatment for at least 4 weeks before exposure. Exclusion criteria included significant comorbidities, uncontrolled asthma, or contraindications to EEC exposure. Relevant demographic and clinical characteristics were recorded at baseline using REDCap (Research Electronic Data Capture; Vanderbilt University).

In total, 34 grass pollen provocations were conducted, as 3 participants (1 control and 2 allergic individuals) underwent 2 separate exposures with a washout period of at least 4 weeks. In addition, 7 placebo exposures were performed (in both controls and allergic individuals) under identical chamber conditions but without allergen administration. No symptoms were observed during these sessions, supporting the specificity of the protocol.

As this was a pilot feasibility and implementation study, no formal sample size calculation was performed. The study was not statistically powered to test hypotheses, but rather designed to assess the usability and integration of the system in a real-world clinical setting. Consequently, randomization was not applied, as the focus was not on clinical outcome comparison but on technical validation.

### Data Collection and Management

#### REDCap

REDCap, developed by Vanderbilt University, is a secure, web-based app designed to support data capture for research studies [7]. In this study, REDCap was used to build the project infrastructure required for collecting and storing patient-provided data in a structured and accessible manner. Its dual-interface design offers functionalities that are tailored to both health care professionals (HCP) and patients, facilitating seamless data entry, remote symptom tracking, and centralized monitoring.

REDCap facilitates the creation of 2 types of forms essential for collecting clinical data. First, forms are completed by HCPs to document patient information during the EEC exposure process. Second, surveys are completed by patients to record their symptoms throughout the process, without requiring access to the project or account creation.

Patients completed 3 types of surveys—before, during, and after exposure. These surveys included standardized questionnaires:Visual Analog Scale (VAS) for nasal and general symptoms, Total Nasal Symptom Score (TNSS) and Total Ocular Symptom Score (TOSS), along with specific asthma questionnaires [8]. In addition, participants recorded objective respiratory measurements, including peak expiratory flow (PEF; Mini Wright, Clement Clarke International Ltd) and peak nasal inspiratory flow (PNIF; In-check, Clement Clarke International Ltd) [9,10].

At the beginning of the process, each patient was assigned a unique record number to identify all associated forms and surveys. Surveys completed multiple times were distinguished by an instance number (eg, record number “1” will have instance numbers “1,” “2,” and “3” for 3 survey completions). Both forms and surveys can be edited by HCP.

The data derived from patients, aimed at determining results and obtaining clinically useful conclusions, align with the concept of “Patient Reported Outcomes” (PROMs). These outcomes represent the patient’s direct perspective on their symptoms, quality of life, and response to treatment, without external interpretation. As part of the study, we included a work package that incorporated clinically validated questionnaires specifically created to identify unmet needs and improve patient health outcomes.

Data collection was organized into “events” grouping forms to be completed by both HCP and patients (via surveys) throughout the EEC exposure process. The process was divided into 7 events to collect and group information before, during, and after exposure. This structure allowed for automated survey distribution to patients at specified times and frequencies.

Reports were configured in REDCap to facilitate the review and export of information collected at each stage of the process. Users can filter data by record number, allowing targeted access to specific patient sessions or time points. The platform supports export in a wide range of formats, including CSV, SPSS (IBM Corp), Stata, SAS (SAS Institute Inc), R, and XML, making the data accessible for various types of statistical and programming tools.

#### Power BI Integration

To enhance data accessibility for medical staff, the collected data from REDCap were integrated into Power BI, a suite of business analytics tool developed by Microsoft for data visualization and sharing insights [11]. This integration enabled the creation of a live “monitor” displaying key variables—such as VAS, TNSS, and TOSS—through customizable filters and a color-coded system that highlights symptom severity. By presenting information in a user-friendly interface, even nontechnical staff could interpret patient status at a glance, improving clinical responsiveness and workflow efficiency during allergen exposure.

To ensure data integrity and reliability, several quality control measures were implemented throughout the digital workflow. REDCap forms were configured with field validation rules and required fields to minimize missing or inconsistent entries. During data transfer, synchronization routines were programmed to flag incomplete or anomalous data, which were subsequently verified against the original REDCap entries. In addition, during real-time monitoring in the EEC, the system was set up to alert health care staff if survey responses were missing or out of the expected range, enabling immediate review and resolution when necessary.

### Ethical Considerations

The study was approved by the Bioethics Committee of RyCUH, with record number 445, dated December 20, 2022. The procedures followed were in accordance with the World Medical Association’s Declaration of Helsinki. Informed consent was obtained from each participant.

## Results

### REDCap Data Collection

**Figure 1.**
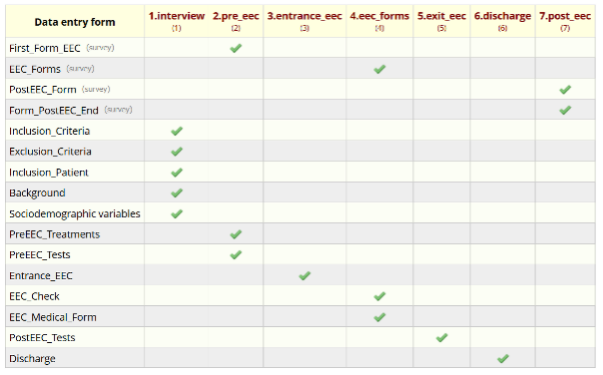
Events in REDCap (Research Electronic Data Capture; Vanderbilt University) and their associated forms. In the left column, all the forms designed to collect data on environmental exposure chamber exposure are represented. The first row contains the names of the events that make up the process. Therefore, the table indicates which forms belong to each event. EEC: environmental exposure chamber.

The REDCap project was structured to align with the procedural stages of patient exposure within the EEC. Seven distinct events were defined, each corresponding to specific phases of the patient’s process, such as clinical interview, entry into the chamber, and postexposure follow-up. Each event was linked to dedicated forms and surveys, ensuring comprehensive and time-aligned data collection, as depicted in Figure 1.

The initial event, “1.interview,” encompasses forms for clinical interview, inclusion and exclusion criteria, patient personal data, background, and sociodemographic variables. These forms are completed by HCPs to assess candidate eligibility for EEC exposure and to collect pertinent clinical and contact information. It is important to note that the patient’s personal data are excluded from data exports to maintain the patient’s anonymity.

The subsequent event, “2.pre_eec,” is completed on the day of the patient’s potential exposure, as it will serve to record treatments or tests carried out during the last 2 weeks that may contraindicate EEC entry. Upon confirming eligibility [6], a baseline symptom survey is emailed to the patient. At this point, the questions included in the survey are explained to the patient, since these will be the same questions that they will answer in subsequent surveys during the exposure. This survey includes assessments such as VAS for general and allergic rhinitis symptoms, TNSS, TOSS, and an asthma symptom questionnaire, as well as measurements of PEF and PNIF. An open-ended section allows patients to report additional symptoms.

Event “3.entrance_eec” records the patient’s entry time into the EEC. This timestamp triggers the automated dispatch of surveys every 15 minutes during the exposure period. This setup enables the collection of up to 6 time-stamped responses per session, allowing for detailed temporal tracking of symptom progression.

Event “4.eec_forms” includes several critical components. It contains patient-completed questionnaires, a form documenting EEC environmental conditions at the beginning and end of the exposure (eg, temperature, pressure, humidity, and airflow), and an optional form activated if severe symptoms require intervention. Completion of this form halts further survey distribution if the exposure lasts less than the planned 90 minutes.

Upon EEC exit, event “5.exit_eec” is completed by HCP to document postexposure assessments and treatments. These include spirometry, fractional exhaled nitric oxide (FeNO; NIOX VERO, Circassia AB), vital signs (blood pressure, heart rate, respiratory rate, and temperature), oxygen saturation, and clinical examinations, with the possibility of recording the results of several spirometries, if necessary. The form also records administered treatments and what it was (eg, antihistamines, corticosteroids, vasoconstrictors, and adrenaline) and determines the provocation outcome in terms of positive or negative for rhinitis, conjunctivitis, or asthma based on symptom scores and predefined thresholds.

Event “6.discharge” logs the patient’s discharge time. This timestamp prompts REDCap to send follow-up surveys every 2 hours on the day of exposure. A final survey is dispatched the following morning, thereby completing the postexposure data collection sequence.

**Figure 2.**
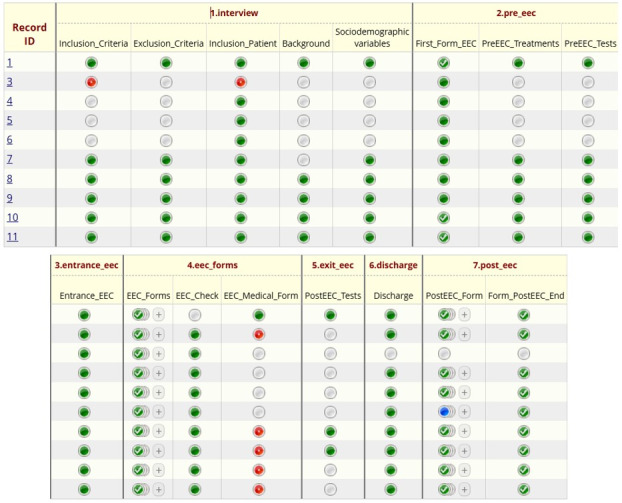
Record Status Console in REDCap (Research Electronic Data Capture, Vanderbilt University). Displays collected records, initial patient exposure events, associated forms or surveys, and their completion status. EEC: environmental exposure chamber.

As shown in Figure 2, the Record Status Console in REDCap provides a comprehensive overview of all data collection instruments used throughout the patient exposure process. It visually displays the forms and surveys linked to each event and indicates their completion status. This interface allows study staff to monitor progress and ensure that no required documentation is overlooked at any stage.

### Patient Monitoring Using Power BI

**Figure 3.**
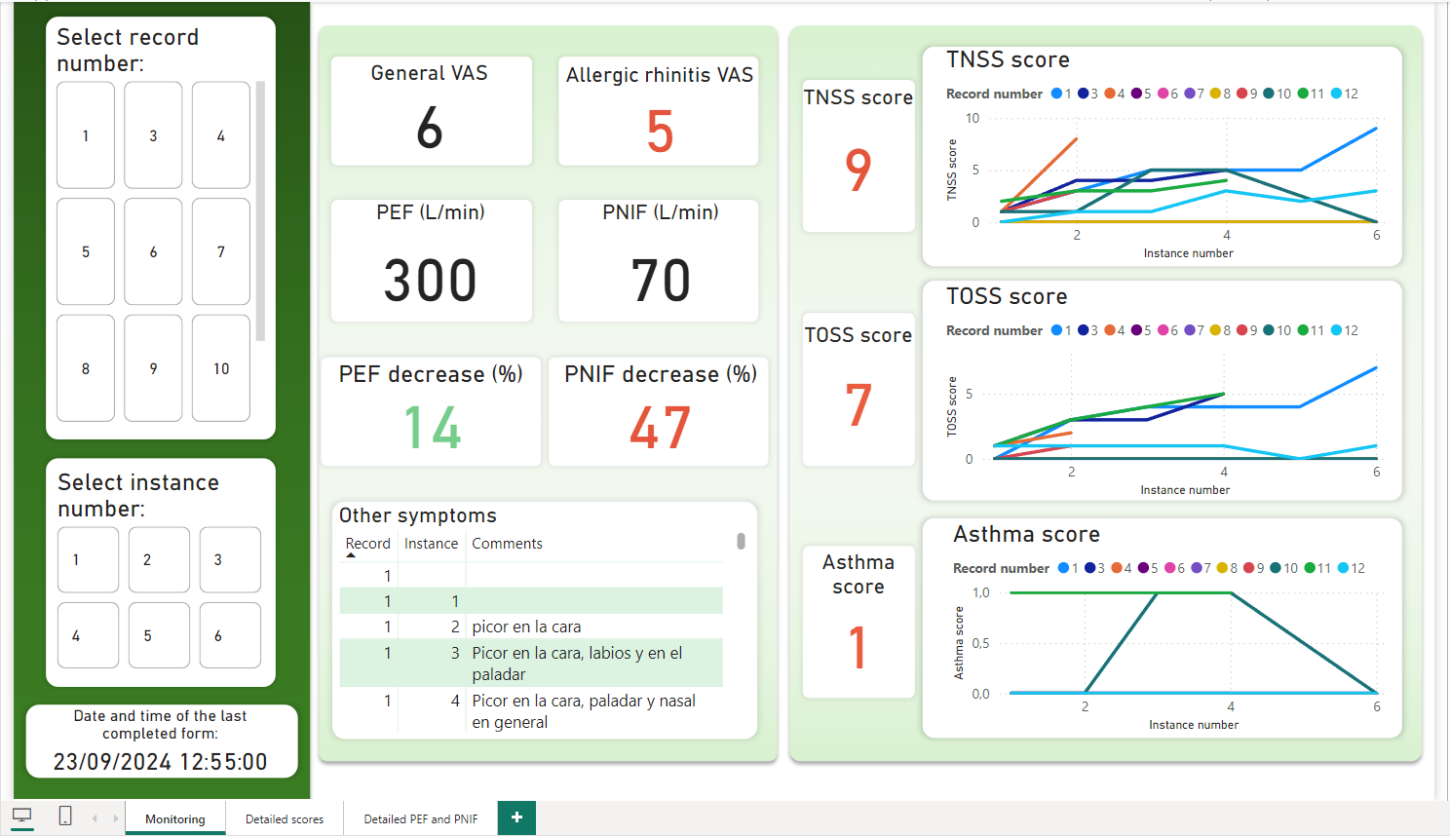
Power BI main dashboard. Illustrates the progression of patient record number 1 across 6 survey instances during environmental exposure chamber exposure. Default values represent the most severe readings, as recorded in instance number 6. EEC: environmental exposure chamber; PEF: peak expiratory flow; PNIF: peak nasal inspiratory flow; TNSS: Total Nasal Symptom Score; TOSS: Total Ocular Symptom Score; VAS: Visual Analog Scale.

REDCap data were successfully integrated into Power BI for comprehensive visualization and monitoring. The primary dashboard (Figure 3) provides a detailed overview of patient status during exposure. A filtering panel on the left allows the study team to select data by record (assigned per EEC entry) and instance number (corresponding to each survey completion). For example, if only the registration number is selected and not the instance number, the evolution of the patient’s symptoms can be seen in the graphs on the right throughout all the registered instances.

The values shown in the labels (“TNSS score,” “General VAS,” “PEF decrease,” and so on) reflect the most severe values recorded within the selected filters. For PEF and PNIF, the lowest values are highlighted, as they indicate the most critical respiratory function readings. The dashboard supports multirecord and multi-instance selections to compare severity across different exposures. In addition, the dashboard indicates the timestamp of the last completed form for each patient. This information is consistently displayed across all dashboard pages.

Certain metrics in the Power BI dashboard are color-coded to indicate the severity of reported symptoms. Acceptable values appear in black or green, while moderate but noncritical readings are highlighted in orange. When values reach a threshold considered severe or potentially hazardous, they are displayed in red, indicating the need for immediate clinical attention.

**Figure 4.**
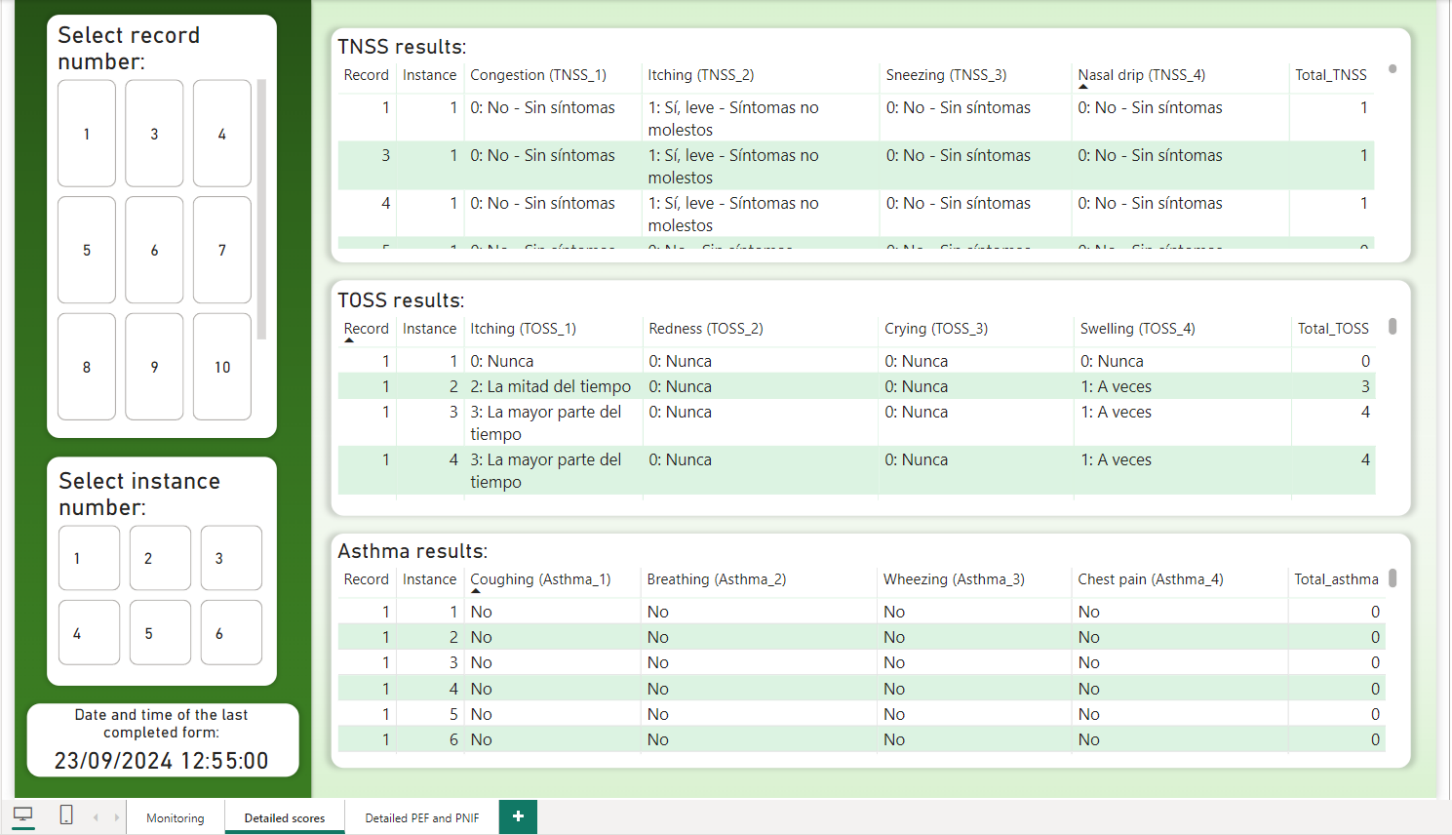
Second page of the Power BI dashboard. Presents raw, detailed responses to the Total Nasal Symptom Score, Total Ocular Symptom Score, and asthma symptoms questionnaire (Spanish is used as the language of the included population) can be observed for each test in each of the tables. Tables include record numbers, instance numbers, questions, and patient responses.TNSS: Total Nasal Symptom Score; TOSS: Total Ocular Symptom Score.

The second dashboard page (Figure 4) offers a general view of patient responses to the TNSS, TOSS, and questionnaires related to asthma symptoms. These results are displayed in detailed tables, each showing record number, instance number, individual questions, and corresponding answers. This structure facilitates precise analysis of symptom patterns and enables comparisons across different time points or patients.

**Figure 5.**
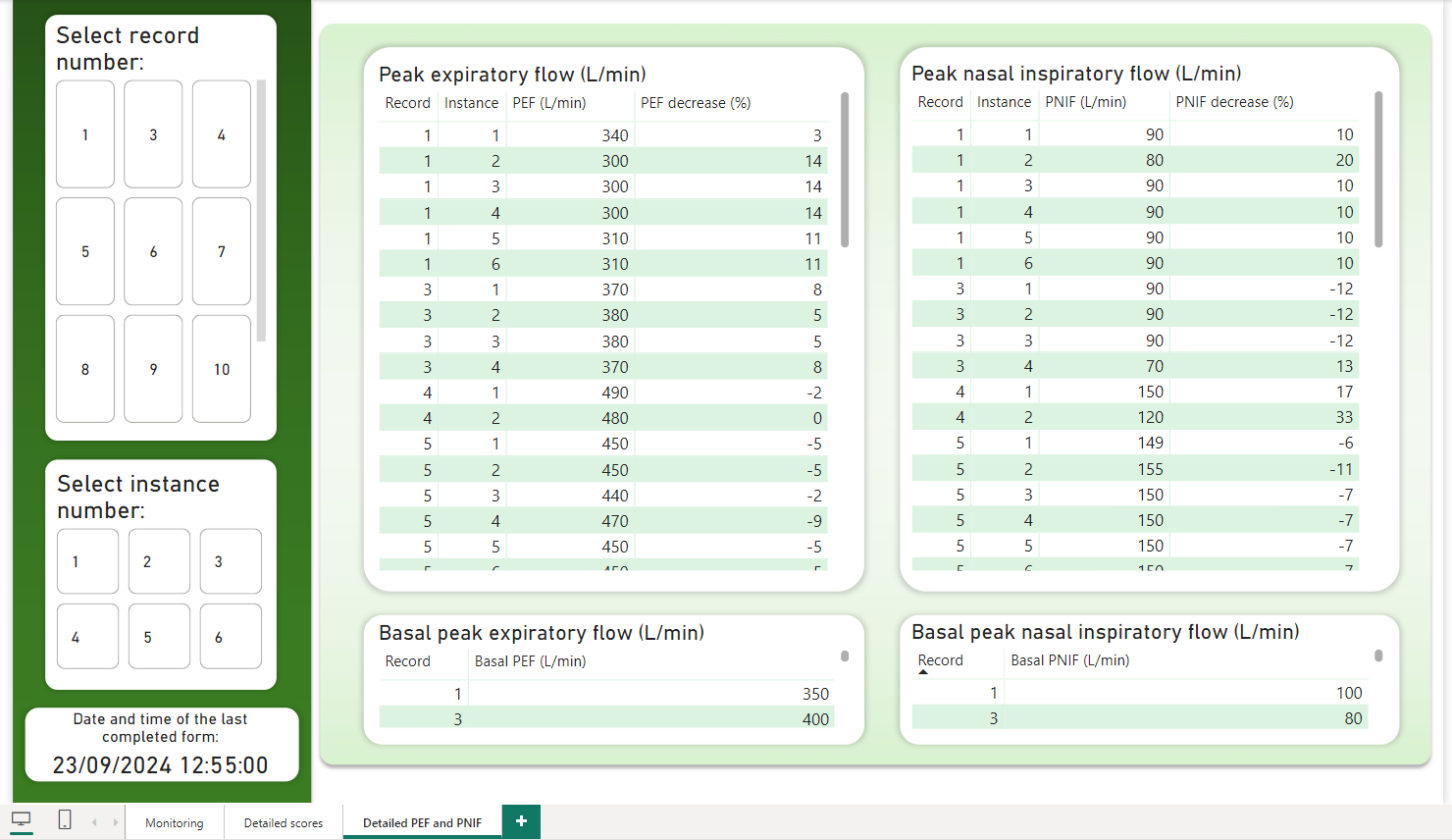
Third and last page of the Power BI dashboard. Displays peak expiratory flow and peak nasal inspiratory flow test results across all instances for record number 1, including baseline values recorded during the initial pre-exposure survey. PEF: peak expiratory flow; PNIF: peak nasal inspiratory flow.

The third dashboard (Figure 5) focuses on PEF and PNIF results. It allows the research team to compare multiple recordings within the same instance or across different moments in the exposure process. This facilitates a more accurate evaluation of the patient’s respiratory function and helps identify potential patterns or deterioration over time. A short video has been added as Multimedia Appendix 1 showing the operation of the EEC using this system.

## Discussion

### Principal Findings

In this study, we developed and implemented a digital monitoring system that integrates REDCap and Power BI to enable structured data collection and real-time visualization during controlled allergen exposures in an EEC. The platform allowed for continuous remote tracking of PROMs, symptom progression, and PEF and PNIF values, facilitating timely clinical decision-making. These results support the feasibility and usefulness of applying digital tools to improve patient monitoring and operational efficiency in allergen challenge studies.

Telemedicine and remote care have emerged as revolutionary forces in the health care sector, transforming the way medical services are delivered and accessed in various specialties, including allergology. These tools have enabled more flexible, scalable, and timely interactions between HCPs and patients, particularly in chronic disease management. With the advancements in technology and the growing demand for efficient health care, telemedicine has become an essential resource for allergists, other HCPs, and patients alike [5].

The specialty of allergology has witnessed significant advances in telemedicine in recent years. Telemedicine in this medical specialty in Spain involves the use of telecommunications and digital technologies to provide internet-based consultations, monitor patients remotely, offer allergy advice, and facilitate follow-up care. This approach has proven to be particularly beneficial for Spanish patients with allergy who often require ongoing management and support [12].

Telemedicine in Spain has emerged as particularly effective in managing asthma and allergic rhinitis, 2 of the most common allergic conditions. Patients can consult with allergists through video calls, receive personalized treatment plans, and learn about environmental control measures to mitigate allergen exposure, or even benefit from the use of connected devices to monitor their lung function and share the data with health care providers [13]. This allows for early detection of asthma exacerbations and timely intervention.

Telemedicine and remote care have had a profound impact on allergy care in Spain, bringing specialized allergy care within reach of patients across the country. However, in certain cases, face-to-face consultations are still required for comprehensive diagnosis and treatment. Integrating telemedicine with traditional health care practices [14] is essential to ensure continuity of care, comprehensive allergy diagnostics and treatment, and to ensure the best disease control. Building trust through internet-based consultations may require extra effort on the part of HCP [15].

Moreover, all forms of telehealth overcome common barriers of time and transportation, making them convenient for patients and HCPs, subsequently trained [5,16,17].

Allergists and other HCPs must remain informed about the rapidly evolving landscape of regulations and reimbursement policies. This is particularly important as telemedicine continues to expand and integrate into mainstream clinical practice. Ensuring compliance with local, regional, national, and international legal frameworks is essential for the safe, ethical, and effective delivery of online care [17].

**Figure 6.**
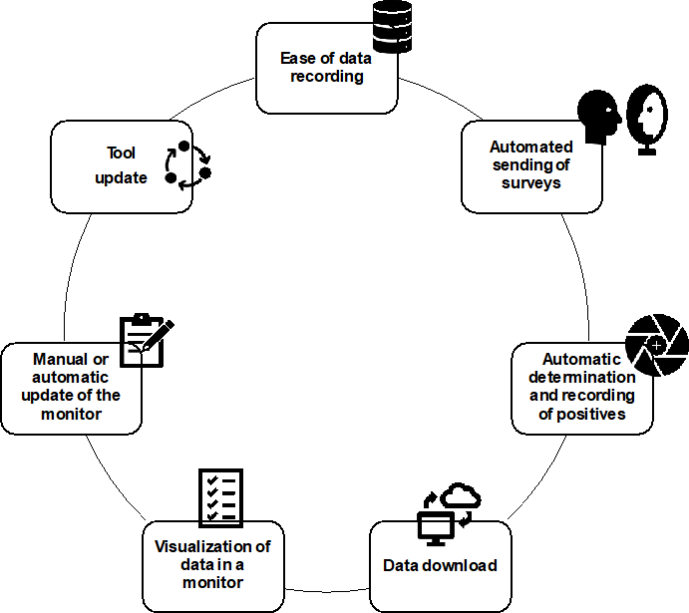
. Characteristics of the innovative eHealth tool for environmental exposure chamber studies.

Telemedicine plays a crucial role in EEC studies, where real-time clinical monitoring is essential and physical interaction is limited. However, there is currently no standardized method for collecting clinical data during EEC provocations [3]. The system presented in this study addresses this gap by offering a versatile and structured digital tool with several practical features (Figure 6): (1) Ease of data recording [5]: the data recording process is structured in different forms, organized in events. This way, medical or research staff have a guide to follow throughout the entire process. As for patients, no training is required to record the data. (2) Automated sending of surveys: medical or research staff do not need to manually configure the sending of any survey; it is enough to record the times of entry to the EEC and discharge of the patient. (3) Automatic determination and recording of positives: for each exposure to the EEC, the form to be filled out with the tests (and possible treatments) of the patient upon leaving the EEC will automatically determine whether the patient presented symptoms of rhinitis, conjunctivitis, or asthma, so that medical staff can fill out the rest of the form later without needing to review the results of the surveys. This point has been especially important during the provocations carried out so far in the EEC in order to avoid errors and to be able to provide the most complete care to the patient [6]. (4) Downloading data in different formats [18]: the collected data can be downloaded in different formats, useful for statistical analysis programs such as SPSS or statistical programming languages such as SAS or R programming language. This is one of the reasons why this study is considered to promote interdisciplinarity, since it offers results that can be analyzed by other professionals related to the medical field. (5) Presentation of the data on a dashboard: as previously mentioned, it was considered necessary to transfer the data to another program to facilitate patient follow-up within the EEC, since the REDCap interface can be complicated for nonspecialized personnel. Thanks to the dashboard that was developed, it was possible to include a color code to indicate the severity of the patient’s symptoms, a visual and striking way for medical or research personnel to identify a possible dangerous situation for the patient. (6) Manual or automatic update of the dashboard: the dashboard can be used in its desktop version, so that the update would be manual (refreshing the dashboard), or it can be uploaded to a server and a time interval configured before the automatic update. (7) Possibility of continuing to update this tool: in case changes are required (eg, more exclusion or inclusion criteria to be checked), both the project in REDCap and the data displayed by the dashboard in Power BI can continue to be updated, which gives the project greater versatility.

The symptoms’ appearance and the measure of scores (TOSS, TNSS, etc) are the main outcomes in the trials searching drug onset of action performed in the EECs [19]. Although it is not yet standardized how the clinical data of patients should be collected in the EEC provocations, using telemedicine is critical in this field [3,20]. All these features of this new infrastructure for data collection during the EEC provocations allow for improved patient follow-up through accurate symptom monitoring, and it is paramount to carry out reliable and accurate EEC studies [6].

Informal feedback from the medical and research staff who participated in the implementation phase has been positive. Users highlighted the clarity and structure provided by the REDCap event design, as well as the real-time visualization in Power BI, which facilitated patient monitoring and reduced the cognitive load during allergen exposure. Several team members expressed that the integration of both tools significantly improved workflow efficiency and confidence in managing symptom progression inside the EEC.

This study has some limitations. It was designed as a pilot feasibility and technical implementation study; therefore, no formal power analysis was performed, and the sample size was not intended for hypothesis testing. The primary objective was to validate the integration and real-time use of the digital health monitoring system in a controlled exposure setting. Furthermore, the study focused exclusively on grass pollen allergy, and the tool has not yet been validated with other allergens or in different EECs. Future studies with larger and more diverse populations are needed to assess broader usability and clinical outcomes.

### Conclusions

This work presents a valuable digital solution for both patients and HCPs involved in allergen exposure studies. By integrating clinical workflows with real-time data monitoring, the system enables comprehensive tracking of PROMs in a controlled environment. Designed collaboratively by experts in clinical care and biomedical research, this tool aims to enhance the safety, precision, and effectiveness of the follow-up of patients with respiratory allergies.

## Supplementary material

10.2196/71276 Multimedia Appendix 1 Short video showing the operation of the implemented system described in the article.

## Abbreviations:

EEC: environmental exposure chamber
FeNO: fractional exhaled nitric oxide
HCP: health care professional
mHealth: mobile health
PEF: peak expiratory flow
PNIF: peak nasal inspiratory flow
PROM: patient-reported outcome
REDCap: Research Electronic Data Capture
RyCUH: Ramón y Cajal University Hospital
TNSS: Total Nasal Symptom Score
TOSS: Total Ocular Symptom Score
VAS: Visual Analog Scale
